# Supplementary material for: ColabFold predicts alternative protein structures from single sequences, coevolution unnecessary for AF-cluster
Source: bioRxiv. 2023 Nov 25:2023.11.21.567977. Preprint. [Version 2] doi: 10.1101/2023.11.21.567977 (PMC10705582; doi:10.1101/2023.11.21.567977)
Supplement: Supplement 1 [file media-1.pdf]

Supplementary Information
ColabFold predicts alternative protein structures from single sequences, coevolution unnecessary for AF-cluster

Lauren L. Porter[1,2,*], Devlina Chakravarty[1], Joseph W. Schafer[1], Ethan A. Chen[1],

[1]National Center for Biotechnology Information, National Library of Medicine, National Institutes of Health, Bethesda, MD 20894
[2]Biochemistry and Biophysics Center, National Heart, Lung, and Blood Institute, National Institutes of Health, Bethesda, MD, 20892

*Correspondence: porterll@nih.gov

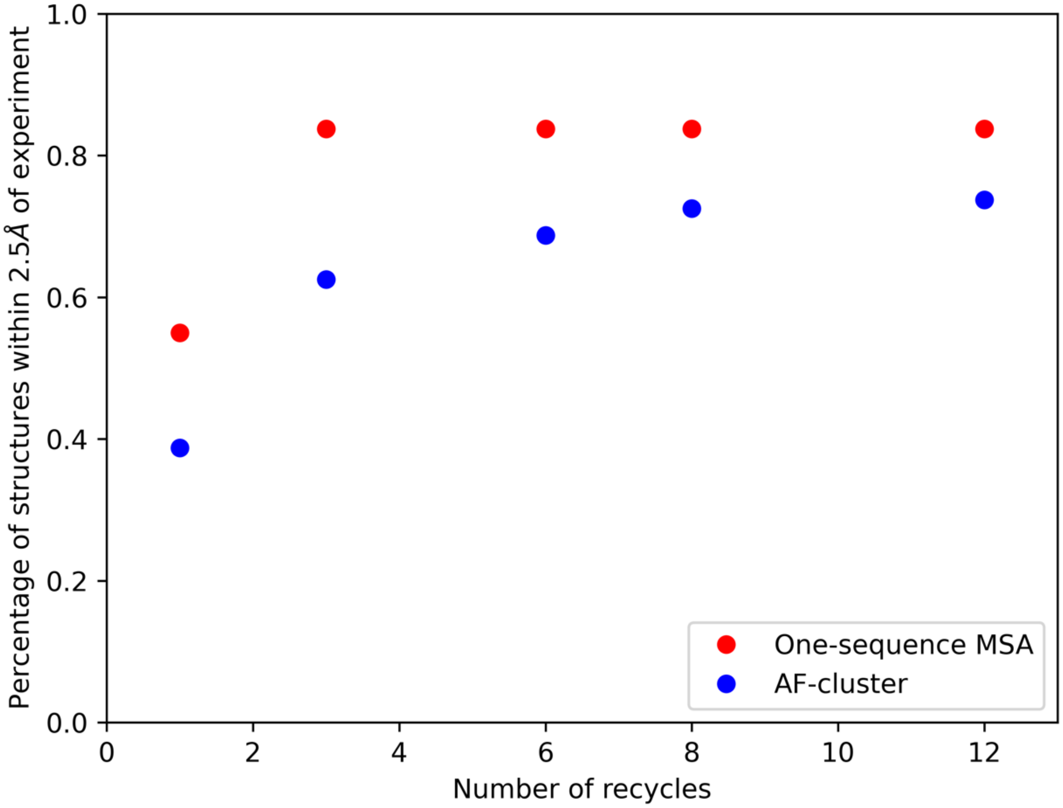

Figure 1. An MSA with one sequence (Uniprot ID: FNY50) enables the sequence of Mad2 to converge to the open conformation faster (with fewer recycles) than the best-performing AF-cluster MSA (1S2H-047.a3m).

Number of recycles	Average RMSD AF-cluster	Average RMSD one-sequence MSA
1	4.4 ± 2.6Å	4.6 ± 4.1Å
3	3.3 ± 2.3Å	2.7 ± 2.7Å
6	2.9 ± 2.1Å	2.1 ± 2.2Å
8	2.7 ± 2.3 Å	2.2 ± 2.1Å
12	2.6 ± 2.3 Å	2.2 ± 2.1Å

Table 1. An MSA with one sequence facilitates more accurate predictions of open Mad2 than the best-performing AF-cluster MSA. Errors are standard deviations from the mean. RMSDs calculated with PyMOL.
